# Effects of Oral Creatine Supplementation on Power Output during Repeated Treadmill Sprinting

**DOI:** 10.3390/nu14061140

**Published:** 2022-03-08

**Authors:** Gregory C. Bogdanis, Mary E. Nevill, George Aphamis, Pinelopi S. Stavrinou, David G. Jenkins, Christoforos D. Giannaki, Henryk K. A. Lakomy, Clyde Williams

**Affiliations:** 1 School of P.E. and Sport Science, National and Kapodistrian University of Athens, 17237 Athens, Greece; 2 Sport, Health and Performance Enhancement (SHAPE) Research Centre, Department of Sport Science, Nottingham Trent University, Nottingham NG11 8NS, UK; mary.nevill@ntu.ac.uk; 3 Department of Life and Health Sciences, University of Nicosia, Nicosia 2417, Cyprus; aphamis.g@unic.ac.cy (G.A.); stavrinou.p@unic.ac.cy (P.S.S.); giannaki.c@unic.ac.cy (C.D.G.); 4 School of Human Movement and Nutrition Sciences, The University of Queensland, Brisbane, QLD 4072, Australia; djenkins@usc.edu.au; 5 School of Health and Behavioural Sciences, University of the Sunshine Coast, Sippy Downs, QLD 4556, Australia; 6 School of Sport, Exercise and Health Sciences, Loughborough University, Loughborough, Leics LE11 3TU, UK; henryk.lakomy@btinternet.com (H.K.A.L.); clyde.williams980@gmail.com (C.W.)

**Keywords:** creatine supplementation, repeated sprints, phosphocreatine, running sprints, blood ammonia

## Abstract

The aim of the present study was to examine the effects of creatine (Cr) supplementation on power output during repeated sprints on a non-motorized treadmill. Sixteen recreationally active males volunteered for this study (age 25.5 ± 4.8 y, height 179 ± 5 cm, body mass 74.8 ± 6.8 kg). All participants received placebo supplementation (75 mg of glucose·kg^−1^·day^−1^) for 5 days and then performed a baseline repeated sprints test (6 × 10 s sprints on a non-motorised treadmill). Thereafter, they were randomly assigned into a Cr (75 mg of Cr monohydrate·kg^−1^·day^−1^) or placebo supplementation, as above, and the repeated sprints test was repeated. After Cr supplementation, body mass was increased by 0.99 ± 0.83 kg (*p =* 0.007), peak power output and peak running speed remained unchanged throughout the test in both groups, while the mean power output and mean running speed during the last 5 s of the sprints increased by 4.5% (*p* = 0.005) and 4.2% to 7.0%, respectively, during the last three sprints (*p =* 0.005 to 0.001). The reduction in speed within each sprint was also blunted by 16.2% (*p =* 0.003) following Cr supplementation. Plasma ammonia decreased by 20.1% (*p* = 0.037) after Cr supplementation, despite the increase in performance. VO_2_ and blood lactate during the repeated sprints test remained unchanged after supplementation, suggesting no alteration of aerobic or glycolytic contribution to adenosine triphosphate production. In conclusion, Cr supplementation improved the mean power and speed in the second half of a repeated sprint running protocol, despite the increased body mass. This improvement was due to the higher power output and running speed in the last 5 s of each 10 s sprint.

## 1. Introduction

In several individual and team sports, training schedules include the performance of repeated sprints once or twice per week in order to improve speed and the ability to repeat intense efforts with short recovery intervals [1,2,3]. The duration of sprints for this type of training ranges between 2 and 10 s, with work/recovery ratios between 1:3 and 1:8 [4]. In sports such as soccer, training with short sprints with 1:6 recovery ratio leads to improvements in repeated sprint performance [3,5], which may be transferred to match performance. Repeated sprint exercise is fuelled mainly by phosphocreatine (PCr), anaerobic glycolysis, and by an increasing aerobic contribution as repetitions progress [6,7,8,9]. Although the major contributor to energy supply in sprints lasting from 6 to 30 s is anaerobic glycolysis, PCr has an increasing role in adenosine triphosphate (ATP) resynthesis when sprints are repeated and especially towards the last part of a sprint [6,7,8,10].

The rapid but incomplete rates of muscle PCr resynthesis during recovery intervals commonly used in sports practice (i.e., 30 s to 3 min) [11] make it very tempting to attempt to alter either PCr resting levels and/or the rate of PCr resynthesis. This is especially important, as the inability to maintain high power output during all-out exercise, i.e., fatigue, has been associated with decreased phosphocreatine levels [8,12], while the rate of recovery mirrors that of power output during the initial seconds of a repeated bout [6]. Early studies [8] reported that phosphocreatine (PCr) is the single anaerobic source during the last of a series of ten 6 s cycling sprints with 30 s rest [6,13]. This would suggest that a possibly faster PCr resynthesis would improve performance towards the last bouts. Collectively, these findings emphasise PCr as being central for maintaining a high ATP-to-adenosine diphosphate (ADP) ratio and thus a high-power output.

Sprint training has been shown to be relatively ineffective in raising baseline PCr levels [14,15], while the rate of PCr resynthesis can be improved by targeting mitochondrial metabolism using aerobic training [16,17], which may alter muscle fibre composition towards a slower phenotype [18]. The decreased metabolic stress due to improved energy availability has also been shown to reduce the magnitude of purine nucleotide loss which occurs during sprinting, which may be evidenced by decreased plasma ammonia [19,20].

Oral creatine (Cr) supplementation may be considered as a fast and effective method to increase both resting PCr and its rate of resynthesis during repeated high-intensity exercise [21,22,23,24]. Due to the increased PCr availability, Cr supplementation reduces the rate of fatigue during repeated high-intensity exercise [25,26,27,28]. However, the question of whether Cr supplementation may reduce the degradation of adenine nucleotides during exercise still remains [22,29]. Several studies examining the effects of Cr supplementation on sprint performance have used cycling as the exercise mode and with bout durations around 30 s [6,7,30,31]. Whereas these studies provided useful information under laboratory conditions, 30 s efforts are not common in sports, and therefore, studies investigating shorter sprints may provide a more relevant information regarding the effects of Cr supplementation in sports practice. A number of studies have shown an improvement in overall cycling peak power output (PPO) over 5 × 12 s [32], and most commonly an increase and maintenance of mean power output (MPO) on repeated sprints of 10–15 s in duration [21,33,34,35].

There are relatively fewer studies using sprint running compared with cycling. Running-based sprint tests included mostly short distance bouts (≈35 m or less, corresponding to <6 s duration), and reported improvements in sprint times [36,37,38], while other studies have shown no effect [22,39]. Studies using repeated sprints with short recovery in soccer players reported improvements over distances of 5 to 15 m [27] or 6 × 35 m [37]. Although sprint running is the most ecologically valid mode of examining the effects of an intervention on performance in a large number of individual and team sports, the results obtained are limited to a single value (i.e., time taken to cover the distance), while the time course of performance within each bout is unknown.

Although many studies have already addressed Cr supplementation, including its effects on sprint performance, a gap still exists in the literature on the power output and speed during running sprints and how these vary over the duration of a single sprint, as well as over an entire session of repeated sprints. Therefore, the aim of the present study was to examine the effects of Cr supplementation on repeated sprint performance on a non-motorised treadmill, to provide, for the first time, detailed information on power output and speed during running sprints. It was hypothesised that a 5-day supplementation on an individualised basis (per kg body mass) will reduce fatigue both within each sprint and between sprints. Moreover, the effects of indirect indices of anaerobic metabolism (blood lactate and pH), purine nucleotide loss (ammonia), and aerobic metabolism (oxygen uptake and heart rate) were measured during and after sprinting, in an attempt to examine potential mechanisms for changes in sprint performance. Finally, the effects of Cr supplementation on maximal oxygen uptake (VO_2_max) and lactate during submaximal exercise were investigated to identify possible effects of Cr supplementation on aerobic metabolism.

## 2. Materials and Methods

### 2.1. Participants

Sixteen male university students volunteered to participate in this study. Their mean (± standard deviation) age, height, and body mass were 25.5 ± 4.8 y, 179 ± 5 cm, and 74.8 ± 6.8 kg, respectively. Inclusion criteria were (a) healthy males aged 19–30 years, (b) involved in recreational sports, and (c) familiar with sprint running. Exclusion criteria were (a) use of tobacco and related products in the past 6 months, (b) participation in a weight loss program, (c) use of dietary supplements and medications during the 6 months preceding the study, (d) history of endocrine or metabolic disorders, and (e) being on a vegetarian or vegan diet. Prior to commencing the study, potential participants were informed in writing about the purpose of the study, any known risks, and the right to terminate participation at will, and each expressed understanding by signing a statement of informed consent. A medical history questionnaire was also completed in the presence of the main investigator, and individuals with medical problems were excluded. The protocol was approved by the Ethics Committee of Loughborough University (1993/LUT), and the procedures were in compliance with the Code of Practice on Investigations involving Human Participants of Loughborough University.

### 2.2. Overview

**Figure 1:**
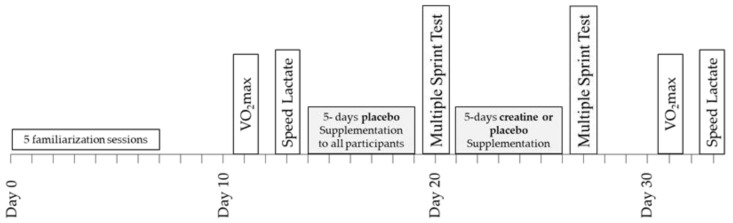
Schematic representation of the experimental design of the study.

A double-blind design was used to study the effects of Cr supplementation on power output, metabolic, and cardiorespiratory responses during repeated sprint running and submaximal exercise (Figure 1). Prior to any testing, all participants were fully familiarised with sprinting on the non-motorised treadmill and running on a motorised treadmill by completing five separate sprinting/running practice sessions including performance of a 6 × 10 s sprinting test with 30 s rest between sprints. The results of this test, which were the same as the main sprint test, were recorded and used to ensure that participants were fully familiarised with the protocol of the study and performed maximally. Four days after the last familiarisation session, a baseline a measurement of VO_2_max was performed, which was followed by a submaximal “speed lactate test” two days later. In the five days following the submaximal test, all participants (*n* = 16) consumed four doses per day of 75 mg glucose·kg^−1^ body mass dissolved in 150–200 mL of fruit juice (orange) at three-hour intervals starting at 9.00 a.m. During these five days, participants followed and recorded their habitual diet and training regimes, with the exception that only very light exercise was performed on day 4, and no exercise was performed on day 5. On the sixth day, the participants arrived at the laboratory at least four hours after their last meal for the baseline sprint session. After completion of the multiple sprint test, the participants were randomly divided into two groups: one group (*n* = 8; Placebo) consumed again four doses per day of 75 mg glucose.kg^−1^ body mass for five days, starting on the same day of the week as the first supplementation (Figure 1). The other group (*n* = 8; Cr) consumed 75 mg creatine hydrate·kg^−1^ body mass + 1 g of glucose four times per day for five days. This regimen of oral Cr supplementation resulted in doses of 22.3 ± 1.8 g Cr·day^−1^, which have been shown to significantly increase the total Cr (Cr + PCr) content of the quadriceps muscle [23]. The Cr and placebo treatments were administered in a double-blind fashion. The pre-recorded dietary and exercise patterns were repeated during the five days of placebo and Cr supplementation. On the sixth day of this supplementation period, participants reported again to the laboratory at the same time of day as in the first multiple sprint test and repeated the six 10 s sprints after the standardised warm-up. The VO_2_max and submaximal running tests on the motorised treadmill were repeated 4 and 6 days after the multiple sprint test (Figure 1). The VO_2_max and submaximal tests, both before and after the supplementation period, were preceded by 24 h of rest. Participants were instructed to follow the same individual pre-recorded diet in the 24 h before the first and the second VO_2_max and submaximal tests.

### 2.3. VO_2_max Test

The participants reported to the laboratory having fasted for at least three hours. Each was first weighed wearing their shorts before a heart-rate monitor (SportesterTM, PE3000, Polar Electro Oy^®^, Kempele, Finland) was attached to the chest. A three-min warm-up between 2.8 and 3.0 m·s^−1^ (≈10.1 and 10.8 km·h^−1^) on the motorised treadmill (Woodway, Waukesha, WI, USA) then followed. The test required each participant to maintain a running speed of 3.2 m·s^−1^ (11.5 km·h^−1^); treadmill elevation for the first three minutes was set at 3.5% and was subsequently increased by increments of 2.5% at three-minute intervals until the participant volitionally fatigued.

Expired air was collected in 200 L Douglas bags during the final 60 s of each three-minute period. Ratings of perceived exertion [40] were collected at each level, and heart rate was recorded at 60 s intervals. On completion of the test, FEO_2_ and FECO_2_ were measured (HR) using gas analysers (Sybron Taylor, Servomex O2 Analyser, model OA570, UK and MSA, Lira Infrared analyzer model 303, Pittsburg, PA, USA), which were calibrated with gases of known composition (Cryoservice Ltd., Worcester, UK). The air evacuated from each Douglas bag was measured using a Dry Gas Meter (Harvard). Oxygen consumption was recorded for later analysis.

### 2.4. Speed Lactate Test

Participants reported to the laboratory in a fasted and fully hydrated state. After being weighed and having a heart rate monitor attached (Sports Tester, PE3000, Polar Electro Oy, Helsinki, Finland), each warmed up by running between 2.2 and 3.0 m·s^−1^ (≈10.1 and 10.8 km·h^−1^) for three minutes. Three minutes of passive stretching followed. This test required participants to run at four (continuous) treadmill speeds. For the 16 volunteers, the initial running speed was between 2.5 and 3.06 m·s^−1^, (≈9 and 11 km·h^−1^) depending on running ability. Then, speeds were increased by fixed increments of 0.56 m·s^−1^ (2 km·h^−1^) every four minutes. Heart rate was recorded each minute, while expired air and capillary blood (20 μL) samples were collected during the final 60 s of each four-minute stage. Perceived exertion was also recorded at each speed. Oxygen consumption (VO_2_) was calculated from expired air analysis using the equipment described above, while capillary blood was analysed for lactate.

### 2.5. Repeated Sprint Test

One hour before the multiple sprint tests, participants reported to the laboratory in a fasted and well-hydrated state. A cannula was positioned in an antecubital vein under local anaesthetic (lignocaine) to allow venous blood (5 mL) to be drawn at rest and at one, five, and 10 min following the final (6th) sprint. From these samples, aliquots of blood were immediately analysed for pH, hematocrit (Hct), and hemoglobin (Hb). Two remaining blood samples (20 μL) were added to perchloric acid, centrifuged, and stored at −70 °C for the later analysis of lactate and ammonia. After providing a resting blood sample, each participant performed a standardised warm-up on the non-motorised treadmill, which included 4 min easy running at 50–60% of peak heart rate, followed by 3 min of stretching and two submaximal 5 s sprints. The repeated sprints protocol was executed 3 min later and involved six 10 s treadmill sprints. Power output was calculated as a product of horizontal force (measured by a force transducer attached to a restraining harness secured to the participant) and the speed at which the participant turned the treadmill belt [41]. Data were stored by an interfaced computer and retrieved on completion of exercise.

Thirty seconds of passive recovery separated each of the six sprints; the participants were instructed to begin jogging 27 s into each recovery period and were given the instructions “Three, two, one GO!” before accelerating to full speed. The power and speed parameters analysed were peak (PPO) and mean power output (MPO) during each sprint, peak and mean running speed during each sprint, MPO and mean running speed during the first 5 s and the last 5 s of each sprint, total work completed during all sprints, total completed in the first 5 s and the last 5 s of all sprints, fatigue index (FI) of power and speed at each sprint, according to the formula: FI = peak power or speed minus power or speed at the last second of the sprint, expressed as a percentage of the peak power or speed.

Expired air was collected during the last three sprints (including the recovery periods) for calculation of oxygen consumption. This expired air sample was analysed as previously described. On completion of the sixth sprint, participants had the restraining harness detached from the force transducer and were assisted to a chair to facilitate post-exercise sampling of venous blood (5 mL) five and ten minutes after the end of the test.

Heart rate was recorded immediately before and after each sprint (highest value attained 5–10 s after sprint) and every minute for the first 10 min of recovery using short range telemetry (Sports Tester, PE3000, Polar Electro Oy, Finland).

### 2.6. Blood Analysis

Lactate was assayed using fluorescence spectrometry (Perkin Elmer LS 45, PerkinElmer Inc., Waltham, MA, USA), while venous blood pH was ascertained using a blood micro system (BMS MK2, Blood Micro System, Radiometer, Copenhagen, Denmark), which had been calibrated to within the expected range of pH values (7.383 ± 0.005 to 6.841 ± 0.005) at 37 °C. Plasma volume changes (PVC) were established using the method described by Dill and Costill [42].

### 2.7. Statistical Analyses

A three-way analysis of variance (ANOVA) with repeated measures (groups x pre-post supplementation x sprint number) was used to examine changes for power output, speed, distance, heart rate, and blood parameters. Changes in body mass, VO_2_max, sprint VO_2_, and total work completed were examined using a two-way ANOVA with repeated measures (groups x pre-post supplementation). Tukey’s post hoc tests were performed when a significant main effect or interaction was obtained (*p* < 0.05) to locate differences between means. Effect sizes were calculated using partial eta squared (η^2^) interpreted as small (0.01 to 0.058), medium (0.059 to 0.137), or large (>0.137) [43]. Pearson correlation coefficient (r) was used to examine relationships between variables. Test–retest reliability was assessed by calculating the intra-class correlation coefficient (ICC) using a two-way mixed model for all dependent variables. Data are expressed as means ± standard deviations (SD). All statistical procedures were performed using SPSS (IBM SPSS Statistics, version 23, Armonk, NY, USA).

## 3. Results

### 3.1. Body Mass, VO2max, and Submaximal Test Results

The two-way ANOVA for body mass revealed a main effect of pre–post supplementation (*p* = 0.035, η^2^ = 0.28) and a pre–post x group interaction (*p* = 0.006, η^2^ = 0.42) with only the Cr group increasing body mass after supplementation by 0.99 ± 0.83 kg (+1.36%, *p* = 0.007). No significant effect on VO_2_max was observed following supplementation (placebo: 60.7 ± 7.8 to 61.9 ± 8.0 mL·kg^−1^·min^−1^; Cr: 61.0 ± 4.1 to 60.4 ± 3.8 mL·kg^−1^·min^−1^).

The three-way ANOVA for VO_2_ and heart rate during the submaximal test showed only a sprint number effect (*p* < 0.001, η^2^ = 0.96 and 0.98, respectively), with no differences between groups or pre–post supplementation. In addition, only a main effect of sprint number was found for blood La during the speed lactate test (*p <* 0.001, η^2^ = 0.87, Table 1). The ICC for maximal and submaximal VO_2_ was 0.97–0.99 (*p* < 0.001). The ICC for maximal and submaximal heart rate was 0.93–0.96 (*p* < 0.001), for blood lactate, it was 0.94–0.99 (*p* < 0.001), and for blood pH, it was 0.74–0.94 (*p* < 0.01).

### 3.2. Power Output and Running Speed Parameters during Repeated Sprinting

**Figure 2:**
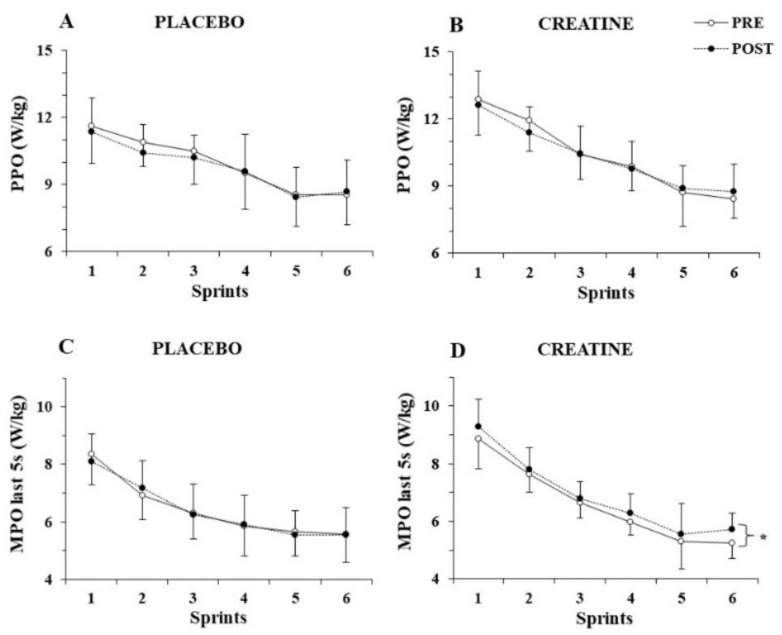
Peak power output (PPO) per kg of body mass (**A**,**B**) and mean power output (MPO) per kg of body mass during the last 5 s (**C**,**D**) of each sprint for the placebo and creatine groups pre- and post-supplementation. * *p* < 0.01 average of all sprints before vs. after supplementation.

The three-way ANOVA for PPO/kg showed a significant main effect of sprint number (*p* < 0.001, η^2^ = 0.82) but no three-way interactions (*p >* 0.77), indicating unchanged PPO/kg before and after supplementation for both groups (Figure 2A,B). The three-way ANOVA for MPO/kg revealed a three-way interaction (group x sprint x pre-post supplementation, *p* = 0.025, η^2^ = 0.16). A post hoc test showed that MPO/kg was unchanged in the placebo group, while it increased in the 6th sprint following Cr supplementation (from 6.0 ± 0.6 to 6.6 ± 0.7; *p* = 0.002).

To further examine the distribution of power output during each sprint, MPO/kg was calculated during the first 5 s and the last 5 s of each sprint and analysed separately. While the three-way ANOVA for MPO/kg achieved during the first 5 s of each sprint did not show any interactions, but rather only a main effect of sprint number (*p* < 0.001, η^2^ = 0.89), the three-way ANOVA for MPO/kg during the last 5 s of each sprint showed a pre–post x group interaction (*p* = 0.010, η^2^ = 0.38), with the post hoc test revealing an average of 4.5% increase in mean power output during the last 5 s of the six sprints after Cr supplementation (*p* = 0.005; Figure 2C,D).

**Figure 3:**
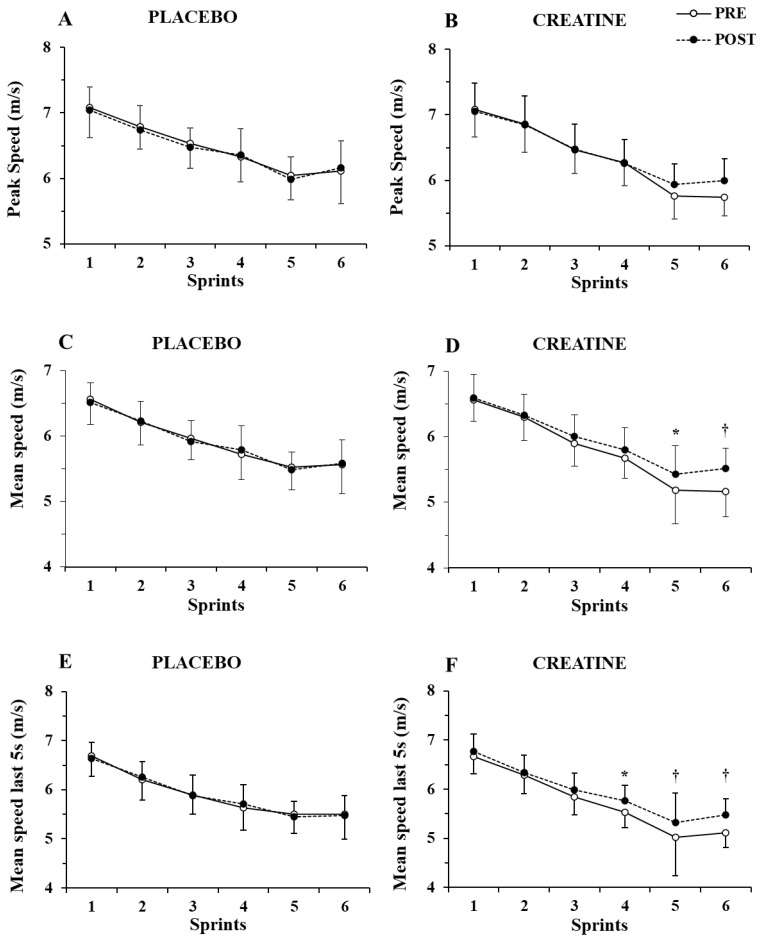
Peak speed (**A**,**B**) and mean speed during the first (**C**,**D**) and last 5 sec (**E**,**F**) of each sprint for the placebo and creatine groups pre- and post-supplementation. * *p* < 0.01; † *p* < 0.001 pre- vs. post-creatine supplementation.

Only a main effect of sprint number (*p* < 0.001, η^2^ = 0.87) without any two-way or three-way interaction was shown by the three-way ANOVA for peak speed (Figure 3A,B). In contrast, a group x sprint interaction (*p* = 0.004, η^2^ = 0.46) and a group x sprint x pre–post supplementation interaction were found for mean speed (*p* = 0.021, η^2^ = 0.17), with the post hoc tests showing 4.6% and 6.9% increase in mean speed in sprints 5 and 6 (*p =* 0.007 and *p <* 0.001, respectively. See Figure 3C,D).

Further examination of mean speed during the first 5 s of each sprint showed only a main effect of sprint number (*p* < 0.001, η^2^ = 0.82) without any interactions. However, the three-way ANOVA of mean speed during the last 5 s of each sprint showed a main effect of sprint number (*p* < 0.001, η^2^ = 0.87) and a group x sprint number x pre–post supplementation interaction (*p* = 0.007, η^2^ = 0.20). A post hoc test showed an increase in mean speed in the last 5 s of sprints 4, 5, and 6 by 4.2%, 6.0%, and 7.0% (*p =* 0.005 to 0.001; Figure 3E,F). The ICC for peak and mean speed was 0.94–0.96 (*p* < 0.001), while for peak and mean power, it was 0.89 to 0.95 (*p* < 0.01).

Fatigue index for power output increased by sprint number (*p* < 0.001, η^2^ = 0.37), with no differences between groups (from 34.2 ± 8.1% in sprint 1 to 40.5 ± 9.1% in sprint 6, *p <* 0.01). However, differences between the two groups (group x pre–post interaction, *p* < 0.017, η^2^ = 0.34) were found for fatigue index for speed, with the post hoc test revealing a decrease in the average fatigue index of all sprints by 16.2% (from 12.7 ± 3.9% before supplementation to 10.6 ± 3.6% after Cr supplementation, *p* = 0.003). There was no change in fatigue index for speed before or after the placebo supplementation (from 12.4 ± 3.8% to 12.1 ± 3.3% before vs. after, *p* = 0.96). This decrease in fatigue index was due to higher speed at the last second of each sprint (group x pre-post interaction, *p* = 0.0014, η^2^ = 0.24).

Total work completed during all 6 sprints was increased by 3.3% after Cr supplementation (from 33,362 ± 3294 to 34,495 ± 4009 J) (*p* = 0.04). The two-way ANOVA for total work completed during the first 5 sec of the sprints also showed no significance. However, analysis for total work completed during the last 5 sec revealed a pre–post x group interaction (*p* = 0.033, η^2^ = 0.29), with the Cr group increasing the total work completed during the last 5 sec after supplementation by 6.0% (*p* = 0.030). Notably, the improvement in total work completed during the entire sprint protocol and during the last 5 s of all sprints was highly correlated with the gain in body mass (r = 0.732, *p =* 0.001 and r = 0.705, *p =* 0.002, respectively).

**Figure 4:**
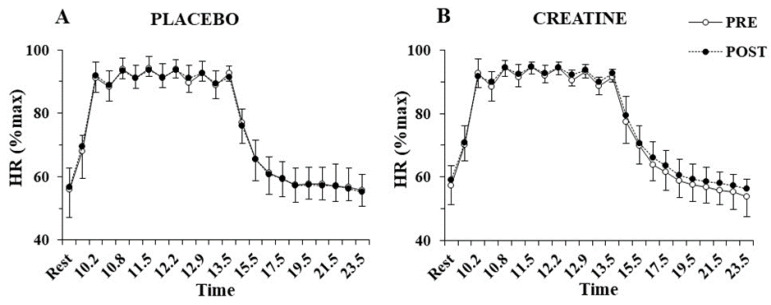
Heart rate (HR) percentage of maximum HR at rest, before and after each sprint, and following 10 min of recovery, pre- and post-supplementation for the placebo (**A**) and the creatine group (**B**).

The three-way ANOVA showed no interactions for heart rate during the sprint tests, indicating no differences between groups before or after supplementation. Only a main effect of sprint number was found for heart rate (*p* < 0.001, η^2^ = 0.97), which fluctuated between 90 and 95% of peak HR during the exercise protocol and recovered to 55% of peak HR after 10 min of recovery (Figure 4). No significant differences were also found VO_2_ during sprints following supplementation in the Cr (70.4 ± 3.3 to 72.8 ± 6.7%VO_2_max; *p* = 0.77) or placebo group (71.7 ± 4.5 to 67.8 ± 6.0%VO_2_max; *p* = 0.20).

**Figure 5:**
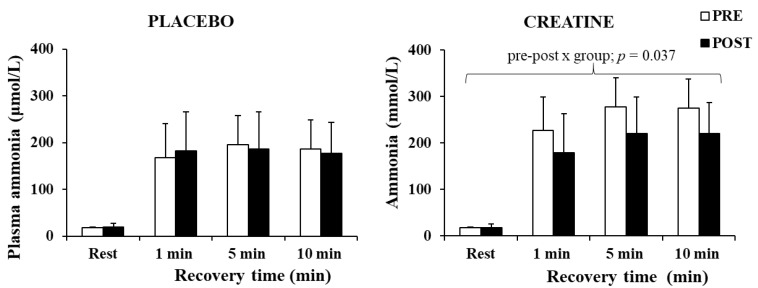
Plasma ammonia at rest and following 1, 5, and 10 min of recovery after the sprint test, pre- and post-supplementation for each group.

The three-way ANOVA for ammonia revealed a significant main effect of time (*p* < 0.001, η^2^ = 0.88) and a pre–post x group interaction (*p* = 0.049, η^2^ = 0.25). A post hoc test revealed a decrease in plasma ammonia by 20.1% after Cr supplementation (*p* = 0.037), without any changes in the placebo group (Figure 5). The decrease in plasma ammonia was negatively correlated with the improvement in the total work completed during the last 5 s of the sprints (r = −0.664, *p =* 0.005).

Only a main effect of time was found for blood La during the sprint (*p <* 0.001, η^2^ = 0.97), with post hoc tests showing a similar increase in La before and after training in the two groups (Table 2). Similar results were obtained for blood pH with only a main effect of sprint number (*p <* 0.001, η^2^ = 0.95; Table 2). A significant main effect of time (*p <* 0.001, η^2^ = 0.83) was also found for PVC with no differences between groups or pre–post supplementation. A post hoc test showed that PVC was highest 1 min post-exercise and was gradually restored during the 10 min recovery (Table 2).

## 4. Discussion

The aim of the present study was to investigate the effects of Cr supplementation on repeated 10 s sprints with a short rest, on treadmill sprint running to provide, for the first time, information on directly measured power output and speed. The present study employed sprint running under controlled laboratory conditions, measuring power output throughout each sprint, thus providing novel data regarding fatigue within and between repeated sprints. Cr supplementation improved sprint endurance and total work completed as reflected by improved MPO and running speed over the second half of the last three sprints. Interestingly, aerobic contribution to energy supply, as implied by VO_2_ measured during the trial, and blood lactate and pH were unchanged after Cr supplementation. On the other hand, plasma ammonia levels after the repeated sprints were 20.1% lower following Cr loading, indicating improved ATP turnover, and reduced adenosine monophosphate (AMP) and inosine monophosphate (IMP) production. Thus, these results imply that Cr supplementation did not affect aerobic and glycolytic metabolism but increased the contribution of PCr, thus reducing metabolic stress despite an increase in mean power and speed.

The improvement of overall performance after Cr supplementation, as reflected by the 3.3% increase in total work over the six sprints, has been reported in other studies on sprinting, using multiple 15 m runs in soccer players [27], 10 s sprints in ice hockey players [44], 6 s [25] and 30 s cycling sprints [7,45]**,** as well as 50-yard swim sprints [46]. The common finding is that the improvement in performance is observed in the latter stages of the exercise session. In the present study, Cr supplementation resulted in better performance during the last 3 sprints, with power output and speed being higher in the last 5 s of each sprint. Importantly, in the present study, which used running as the exercise mode, the increase in body weight of ≈1 kg did not hinder performance, as MPO per kg body mass and average running speed were improved. This would enable practitioners to increase running distance by 4–5%, which would improve session quality and would increase training load without causing greater metabolic perturbations.

The results on power output measurements during the sprints showed that PPO was similar between conditions. PPO during a sprint is achieved during the first 1–2 s of the sprint [30,47,48], where ATP is derived mainly from PCr [8,49]. Even though PCr stores are possibly greater following Cr loading [23,45,50], the rate of creatine kinase reaction would not change, and this might explain the similarity between conditions on PPO [51]. During repeated sprints of short (10 s) duration and recovery (30 s) as in the present study, PCr resynthesis is incomplete (around 69%) [11]. However, this may be adequate to generate PPO even with partial PCr resynthesis, while MPO may require all PCr available in subsequent sprints [10,35].

The increased power output in the Cr group during the latter part of the session could be attributed to an improved PCr resynthesis during the last three sprints, when PCr availability is limited [10,35]. During a 10 s sprint, the muscle PCr level drops by ≈55% [10]. Immediately after, PCr resynthesis is not as fast as previously thought, with a half-time of ≈56 s [6], and even after 6 min, PCr levels do not recover more than 85% of resting level [6,11,13]. Therefore, in the present study, PCr recovery during the 30 s rest between sprints was possibly incomplete; hence, each subsequent sprint was initiated with reduced muscle PCr levels. The mechanism by which Cr supplementation delayed fatigue [26] allowing exercise intensity to be maintained at a higher power output [13,35] was possibly an augment PCr resynthesis during the recovery intervals, especially in the later part of the test [52].

Blood lactate and pH were unchanged by supplementation, suggesting that anaerobic glycolysis remained unaltered by Cr supplementation. Some studies using similar 10-s or longer sprints have reported even lower lactate levels following Cr supplementation [25,53], which in vitro has been shown to be related to increased AMPK activation [54]. During maximal efforts such as sprinting, glycolysis is initiated at the onset of exercise [55], and after 6–10 s, the majority of ATP is derived from glycolysis [7]. For example, ATP derived from glycolysis during a 10 s sprint is around 44% of total ATP production [56]. Since there was no difference in blood lactate, it is safe to assume that in the present study, an increased muscle PCr in the Cr group could have contributed to the increased ATP production during the latter sprints of the session. As shown in previous studies [8], anaerobic glycolysis is almost completely inhibited at the last of ten 6 s cycling sprints, and therefore, energy production is fuelled almost exclusively by PCr and aerobic metabolism. Measurements of VO_2_ during the repeated sprint sessions provide another strong indication that the improved performance of the Cr group was derived from PCr metabolism because there were no differences between the aerobic contributions within the two groups. Similar VO_2_ was also observed during the “speed lactate test”. Other studies have also shown that Cr supplementation does not increase performance and oxygen uptake during aerobic exercise [21,35,57,58], because Cr loading does not influence or alter fuel selection, as estimated by respiratory quotient [21,59].

Importantly, an increase in power output and speed in a weight-bearing activity such as sprint running occurred despite an increase in body weight. Furthermore, the higher power output was accompanied by a large reduction in plasma ammonia (Figure 5), supporting the assumption that the increased ATP turnover did not augment but rather reduced metabolic stress due to the energy derived from increased PCr availability. Ammonia levels are considered an indicator of exercise stress [60], as the transient imbalance between ATP and ADP during muscle contractions may lead to ATP degradation to AMP, which is then metabolised to IMP-producing ammonia [61]. This metabolic pathway and ammonia production appear to intensify in conditions of low PCr [62]. In the present study, ammonia levels in the Cr group were ≈20.1% lower compared with placebo supplementation. This suggests less ADP metabolism to AMP, IMP, and ammonia, due to a possibly quicker ADP rephosphorylation to ATP. This may be a result of, or associated with, the increased muscle PCr levels following Cr supplementation. Greenhaff et al. [24] also reported decreased ammonia production following Cr supplementation, and Casey et al. [45] reported a decrease in ATP loss and concomitantly an increase in work capacity over 30 s maximal isokinetic cycling, suggesting an improved ADP phosphorylation resulting from PCr availability in the muscles. Overall, Cr loading and the improved PCr resynthesis between sprints could reduce ADP accumulation and AMP production. This physiological mechanism would lead to reduced glycolytic rate [25,63] and hence the observation of similar lactate values between conditions in the present study, despite a higher power output during the Cr trial.

Body mass increased in the Cr group, and this was anticipated as it is in accordance with previous studies [21,25]. Following Cr ingestion, the muscle uptake of Cr increases muscle osmolality, leading to acute water entry into the muscle in order to maintain homeostasis, hence the observed rise in body mass due to increases in intracellular and total body water [64,65] without any changes in extracellular body water [64]. A correlation between body weight gain and power output was revealed by the statistical analysis and may indicate that those who increase their body mass during such short-term supplementation protocols [23] exhibit a concomitant increase their performance. Thus, an increase in body mass may be considered as an indirect marker that Cr supplementation would bring about the desired results. It should be noted that no side effects of Cr supplementation were reported in the present study.

The present study has certain limitations. Muscle biopsies were not taken, in order to demonstrate the relationships between performance enhancement and change in muscle metabolism (e.g., increased PCr concentration and faster PCr resynthesis). In addition, body composition was not examined, and thus, the increase in body weight following Cr supplementation may be attributed either to increased muscle mass or water retention. Another limitation is the inclusion of male individuals only. It has been suggested that females may have lower muscle Cr concentration and that they may be less receptive to Cr supplementation due to hormone-related changes in Cr metabolism [66].

## 5. Conclusions

In conclusion, Cr supplementation improved mean power, mean speed, and fatigue index during a series of running sprints, while peak power and peak speed remained unaltered. Increases in power output following Cr loading were observed mainly in the last three 10 s sprints and especially during the last 5 s of each sprint. Changes in sprint running performance occurred despite an increase in body mass and were not accompanied by increase aerobic or glycolytic metabolism, as suggested by indirect measurements of VO2, blood lactate, and pH and PVC. The blunted plasma ammonia response suggests the beneficial effects of Cr supplementation on reducing the extent of energy imbalance during high-intensity exercise. These findings may have a practical effect on athletes of various sports who perform repeated sprints during competition and training or other athletes who perform one last sprint to the finish line during a race. Importantly, Cr supplementation does not improve peak power or speed but contributes to the maintenance of power and speed during the last part of the sprint, thus providing an extra edge to the sports person.

## Figures and Tables

**Table 1:** Blood lactate (La) at rest and at the four stages of the submaximal test, pre- and post-supplementation in each group.

	La (mmol/L)			
	Placebo		Creatine	
	PRE	POST	PRE	POST
Rest	1.2 ± 0.4	1.0 ± 0.2	1.1 ± 0.3	1.1 ± 0.2
Stage 1	2.3 ± 0.8	2.3 ± 0.9	2.5 ± 1.1	2.7 ± 0.8
Stage 2	2.7 ± 0.9	2.8 ± 1.2	3.2 ± 1.3	3.5 ± 1.1
Stage 3	4.2 ± 1.4	4.2 ± 1.9	5.0 ± 2.2	5.4 ± 2.1
Stage 4	7.0 ± 2.3	6.7 ± 2.7	8.3 ± 3.1	9.1 ± 3.0

**Table 2:** Blood metabolites and plasma volume changes (PVC) pre- and post-supplementation in each group at rest and following 1, 5, and 10 min of recovery.

		La (mmol/L)		pH		PVC (%)	
		Placebo	Creatine	Placebo	Creatine	Placebo	Creatine
PRE	Rest	0.8 ± 0.2	0.9 ± 0.3	7.37 ± 0.02	7.37 ± 0.02		
	1 min	13.4 ± 2.6	14.8 ± 1.8	7.05 ± 0.08	6.95 ± 0.07	−12.9 ± 3.3	−16.2 ± 3.7
	5 min	15.2 ± 2.8	17.1 ± 1.7	7.06 ± 0.07	6.98 ± 0.05	−10.4 ± 3.5	−13.8 ± 3.3
	10 min	15.4 ± 3.1	17 ± 1.8	7.08 ± 0.09	6.99 ± 0.06	−8.5 ± 3.1	−11.7 ± 3.5
POST	Rest	1.2 ± 0.9	0.8 ± 0.3	7.36 ± 0.05	7.39 ± 0.04		
	1 min	14.5 ± 2.6	15.4 ± 2.1	7.04 ± 0.11	6.98 ± 0.08	−14.6 ± 7.2	−13.3 ± 4
	5 min	15.5 ± 2.6	17.6 ± 1.5	7.07 ± 0.1	7.0 ± 0.06	−11.4 ± 8.1	−11.2 ± 4.2
	10 min	15.2 ± 3.2	17.7 ± 2	7.09 ± 0.11	7.01 ± 0.06	−9.5 ± 7.5	−9.1 ± 5

## Data Availability

Data will be available on request to the corresponding author.
